# Supplementary material for: TNF-Block Genotypes Influence Susceptibility to HIV-Associated Sensory Neuropathy in Indonesians and South Africans
Source: Int J Mol Sci. 2020 Jan 7;21(2):380. doi: 10.3390/ijms21020380 (PMC7014294; doi:10.3390/ijms21020380)
Supplement: Supplementary file 1 [file ijms-21-00380-s001.pdf]

Table S1. Logistic regression modelling identifies demographic and clinical variables independently associating with HIV-SN in Africans and Indonesians.

Variable	Odds Ratio	P Value	95% CI
**African Optimal Model:** n = 71[a], p = 0.0007, Pseudo R^2 = 0.18			
Weight (kg)	1.04	0.03	1.00–1.08
History of Tuberculosis	4.26	0.07	0.90–20.03
Nadir CD4 T-cells/μL	1.00	0.03	0.99–1.00
**Indonesian Optimal Model:** n = 195[a], p = 0.0006, Pseudo R^2 = 0.08			
Current CD4 T-cells/μL	1.00	0.01	0.99–1.00
>500 copies HIV RNA/mL	3.80	0.04	1.10–13.15

[a] excluding samples with missing demographic, clinical and/or genotype data.
